# Bladder Melanosis: Clinicopathologic Insights Into a Rare Mimic of Malignant Melanoma

**DOI:** 10.1155/crip/5166142

**Published:** 2026-07-03

**Authors:** Mukund Tinguria

**Affiliations:** ^1^ Department of Pathology and Laboratory Medicine, Brantford General Hospital, Brantford, Ontario, Canada, bchsys.org; ^2^ Department of Pathology and Molecular Medicine, McMaster University, Hamilton, Ontario, Canada, mcmaster.ca

**Keywords:** differential diagnosis, malignant melanoma, melanin, melanosis vesicae, spina bifida, urinary bladder, urothelium

## Abstract

Melanosis of the urinary bladder (melanosis vesicae) is a rare condition where dark, velvety patches are seen on the bladder mucosa during cystoscopy. With fewer than 30 cases reported to date, it remains an exceptionally rare finding. The condition is often associated with symptoms like urinary urgency or incontinence. Microscopically, the diagnosis is confirmed by the presence of melanin granules within urothelial cells and lamina propria macrophages. Although the exact cause is unknown, melanosis is considered benign. However, its clinical importance lies in the need to distinguish it from primary or metastatic malignant melanoma. It must also be differentiated from other forms of bladder pigmentation, such as hemosiderin or lipofuscin deposition. Because it can mimic a malignancy, urologists and pathologists must be aware of this entity to avoid an incorrect diagnosis of cancer. This report describes a case of melanosis vesicae in a 26‐year‐old male with spina bifida who presented with lower urinary tract symptoms and a suspected bladder tumor. The clinicopathologic features and a review of the current literature are provided.

## 1. Introduction

Melanosis is defined as the abnormal deposition of melanin pigment within tissues, typically in the absence of melanocytes. When this occurs in the lower urinary tract, it is known as melanosis vesicae, a rare condition characterized by melanin deposits in the bladder urothelium. Since the first reported case in 1986, fewer than 30 instances have been documented in the literature [1, 2]. Patients generally present with nonspecific lower urinary tract symptoms (LUTS), including urgency, incontinence, hematuria, or cystitis. Histologically, the diagnosis is confirmed by the presence of melanin granules within urothelial cells or lamina propria macrophages, which may or may not be accompanied by cytologically bland melanocytes.

Although the etiology of bladder melanosis remains obscure, it is regarded as a benign condition. Its primary clinical importance lies in the differential diagnosis, as it must be distinguished from primary or metastatic malignant melanoma. It should also be differentiated from other forms of intramucosal pigmentation, such as hemosiderin or lipofuscin deposition. Currently, the clinical significance of melanosis vesicae is not well‐defined, and there are no established management protocols [3]. This report presents a case of melanosis vesicae in a 26‐year‐old male with spina bifida who presented with LUTS and a clinical suspicion of a bladder tumor.

## 2. Case Presentation

### 2.1. Clinical History and Cystoscopic Findings

A 26‐year‐old male with a known history of spina bifida presented with chronic LUTS, primarily characterized by urinary urgency, increased frequency, and urinary incontinence. These clinical findings were highly suggestive of a neurogenic bladder secondary to his underlying spina bifida. There was no documented history of chronic clean intermittent catheterization (CIC), indwelling catheter use, or recurrent symptomatic urinary tract infections. Previous clinical management for his neurogenic bladder had been conservative.

Diagnostic cystoscopy revealed moderate trabeculation of the bladder wall and a severely reduced bladder capacity. The bladder mucosa exhibited diffuse, marked, dark‐brown to black velvety pigmentation, which heavily obscured the visualization of both ureteric orifices. No discrete exophytic bladder masses or papillary lesions were identified. Given the striking mucosal pigmentation, the clinical differential diagnosis included melanoma and melanosis vesicae. Multiple cold‐cup mucosal biopsies were performed for definitive pathologic evaluation.

### 2.2. Pathologic Findings

**Figure 1:**
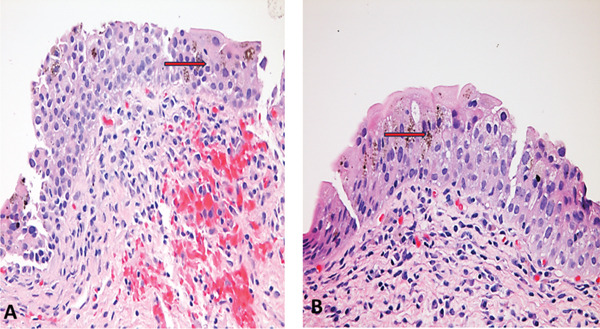
Biopsy of the bladder mucosa showing fragments lined by benign urothelium with reactive changes (A). Abundant granular, dark brown intracytoplasmic pigment is noted within the urothelial cells and underlying lamina propria macrophages (indicated by arrows) alongside a moderate chronic inflammatory infiltrate within the stroma (B) (hematoxylin and eosin, 200× magnification).

The biopsy specimen consisted of multiple fragments of dark‐tan tissue measuring 0.7 × 0.7 × 0.3 cm in aggregate. Histologic examination revealed bladder mucosal fragments lined by urothelium showing reactive changes and abundant, intracytoplasmic, granular, golden‐brown pigment. The underlying lamina propria demonstrated a moderate chronic inflammation with scattered lymphoid aggregates (Figure 1).

**Figure 2:**
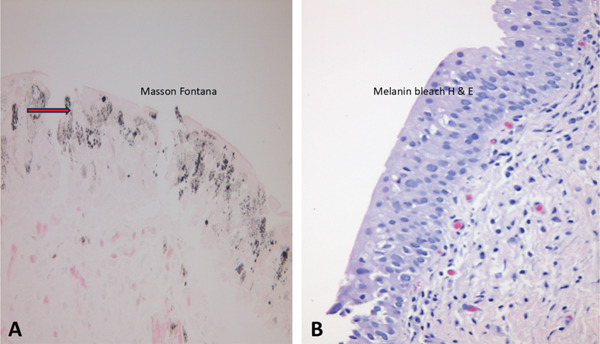
Histochemical characterization of the intramucosal pigment. (A) Fontana–Masson silver stain demonstrating strong focal positivity highlighting the intracytoplasmic granules (200× magnification). (B) Complete absence of silver staining following the melanin bleaching procedure, confirming the presence of true melanin pigment (200× magnification).

**Figure 3:**
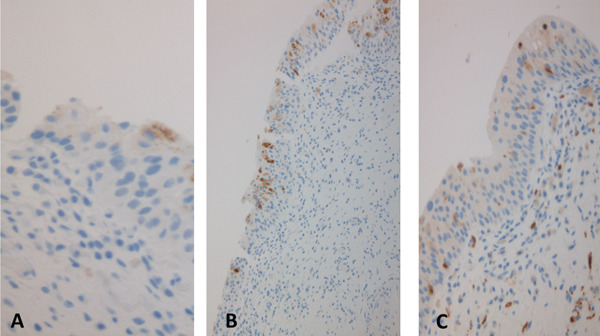
Immunohistochemical profile of the bladder mucosal biopsy. (A) Cytokeratin 20 (CK20) expression is normally restricted to the superficial umbrella cell layer (400× magnification). (B) Immunohistochemical staining for S100 is entirely negative within the urothelial cells and the lamina propria, ruling out a melanocytic proliferation (200× magnification). (C) Negative immunoreactivity for the melanoma‐specific cocktail marker (HMB‐45, Melan‐A, and tyrosinase), confirming the absence of malignant melanoma cells (200× magnification).

To characterize the pigment, histochemical staining was performed. The Fontana–Masson silver stain strongly highlighted the intracytoplasmic pigment within the urothelial cells and superficial lamina propria macrophages. Melanin bleaching successfully removed the silver staining, confirming the pigment as melanin (Figure 2). Prussian blue iron staining was entirely negative, ruling out hemosiderin deposition. Immunohistochemical analysis demonstrated normal, restricted cytokeratin 20 (CK20) positivity in the superficial umbrella cells. Melanocytic and melanoma‐specific markers—including S100, HMB‐45, and a melanoma cocktail (comprising HMB‐45, Melan‐A, and tyrosinase)—were entirely negative, ruling out a melanocytic proliferation or malignant melanoma. The combined morphologic, histochemical, and immunohistochemical features confirmed a diagnosis of bladder melanosis with no evidence of malignancy (Figure 3).

### 2.3. Management and Follow‐Up

Following the benign pathologic diagnosis, the patient was managed conservatively with a focus on symptom optimization for his neurogenic bladder. The patient remains under regular urological surveillance, with a plan for clinical follow‐up and repeat surveillance cystoscopy at 12 months to monitor the mucosal changes and screen for any structural progression.

## 3. Discussion

Melanosis, defined as the abnormal deposition of true melanin within mucosal or epithelial surfaces, is a well‐documented phenomenon in sites derived from ectodermal layers, such as the oral cavity and conjunctiva [4]. In contrast, its occurrence within the endodermally derived genitourinary tract remains an exceptional rarity [5, 6]. Historically, reported cases of melanosis vesicae have been confined to older adults, typically between the ages of 43 and 86 years, with a recorded male predominance [5]. The presentation of this condition in a 26‐year‐old patient represents a significant deviation from established clinical patterns, making this case one of the youngest documented in the literature. Patients generally present with nonspecific LUTS, including gross hematuria, dysuria, urgency, and urinary incontinence. Whether this pigmentary change directly induces voiding dysfunction or represents an incidental finding uncovered during endoscopic evaluation for unrelated urological complaints remains an open clinical question [7].

Cystoscopic evaluation typically reveals distinct, dark‐brown to black, flat or velvety mucosal patches distributed multifocally throughout the bladder. Histologically, these areas correspond to the accumulation of fine, granular, golden‐brown intracytoplasmic pigment concentrated within the urothelium [4, 5, 8–10] and tracking into the cytoplasm of superficial lamina propria macrophages [5, 8]. Despite the heavy pigmentation, the structural architecture of the urothelium remains intact, retaining a normal maturation sequence and a preserved superficial umbrella cell layer. The primary differential diagnosis of a heavily pigmented bladder mucosa includes primary or metastatic malignant melanoma, which must be rigorously excluded; some authors have posited that melanosis may represent a precursor lesion [6, 8]. Malignant melanoma exhibits prominent cytological atypia, architectural disorganization, mitotic activity, and robust immunohistochemical expression of melanocytic markers such as S100, Melan‐A, SOX10, and HMB‐45 [11–13].

Beyond malignant neoplasms, true melanosis must be histologically differentiated from lipofuscinosis and hemosiderin deposition. Lipofuscin, a wear‐and‐tear pigment associated with aging and lipid peroxidation, typically forms fine perinuclear granules that react strongly with a periodic acid‐Schiff (PAS) stain but remain negative on Fontana–Masson and Prussian blue reactions. Additionally, lipofuscin is completely resistant to chemical bleaching procedures. Hemosiderin, arising from erythrocyte breakdown, displays a coarse, refractile, golden‐yellow appearance and is selectively identified via Perl′s Prussian blue reaction. In the present case, a definitive diagnosis of melanosis vesicae was established by strong positivity with a Fontana–Masson silver stain, complete clearing of the pigment following a melanin bleach protocol, a negative Prussian blue stain, and a lack of immunoreactivity for melanocytic markers.

The biological mechanisms governing the development of bladder melanosis remain a subject of active speculation. Early developmental hypotheses centered on two distinct concepts: the anomalous embryonic migration of ectodermal neural crest cells to the lower urinary tract or the directed melanocytic differentiation of multipotent urothelial stem cells [4]. However, because true melanocytes are consistently absent in the vast majority of bladder biopsies, these classical theories face significant scrutiny. Alternative mechanisms suggest that localized, chronic mucosal irritation or neurogenic bladder dysfunction might drive abnormal cellular plasticity or trigger an inductive response from the local mucosal nerve plexus. Pandian et al. [14] suggested a third possible process, melanuria, noting that the melanin appeared to be within lysosomes rather than melanosomes. If that were the case, melanosis of the bladder might be commoner in dark‐skinned races than in white Caucasians, though available demographic data indicate Caucasians represent 80%, and Afro‐Caribbeans represent 20% of subjects where race is reported [14]. In this particular case, the patient′s underlying spina bifida—a major neural tube defect—may provide a unique developmental context. Congenital neuroectodermal anomalies could theoretically disrupt regional cell migration pathways or predispose the pelvic organs to aberrant neuromucosal interactions, fostering microenvironmental conditions that support urothelial melanin accumulation.

The long‐term prognostic trajectory and biological behavior of melanosis vesicae are poorly defined due to a historical scarcity of long‐term data, with the longest reported follow‐up being 10 years [15]. The condition is generally regarded as a benign, indolent, and incidental process with no firmly established connection to malignancy [3]. Complete spontaneous resolution of the abnormal discoloration has been demonstrated in follow‐up cystoscopy 1 year after the initial diagnosis [16]. However, recent literature spanning 2023–2026 has expanded our understanding of concurrent pathologies. Although a direct causal mechanism linking melanosis to neoplastic transformation has not been proved, synchronous presentations of melanosis vesicae alongside high‐grade urothelial carcinoma and malignant melanoma have been reported in up to 16% of tracked cases [3, 17]. This clinical overlap raises the question whether a shared microenvironmental stimulus, such as chronic inflammation or tissue remodeling, may simultaneously predispose the mucosa to both melanosis and urothelial malignancy [3, 17]. Given these documented synchronous findings, the benign histological nature of a primary biopsy should not engender clinical complacency. Regular surveillance cystoscopy and targeted biopsy protocols remain necessary to monitor structural changes and detect early malignant development.

Further studies and long‐term follow‐up are needed to determine the precise relationship between melanosis of the bladder and urothelial carcinoma or malignant melanoma.

## 4. Conclusion

Bladder melanosis remains an exceptionally rare clinical and pathological entity that mimics primary or metastatic malignant melanoma during cystoscopic examination. Accurate diagnostic identification is critical to prevent aggressive, unnecessary surgical overtreatment. Although the condition is benign in the absence of melanocytic atypia or synchronous epithelial lesions, its long‐term biological behavior is not fully defined. Consequently, regular surveillance cystoscopy paired with targeted biopsies represents a prudent monitoring strategy. Pathologists and urologists should maintain clinical awareness of this condition when evaluating dark, pigmented mucosal lesions. Further investigative efforts and centralized data collection are needed to elucidate potential associations with concurrent lower urinary tract pathologies, chronic voiding dysfunction, or urothelial malignancies.

## Funding

No funding was received for this manuscript.

## Consent

This case report has been completely anonymized, and all tissue was obtained as part of the standard of care for the patient; hence, no consent was required.

## Conflicts of Interest

The author declares no conflicts of interest.

## Data Availability

Data sharing is not applicable to this article as no datasets were generated or analyzed during the current study.
